# The role of curcumin on apoptosis and NLRP3 inflammasome-dependent pyroptosis on colorectal cancer in vitro

**DOI:** 10.55730/1300-0144.5652

**Published:** 2023-03-27

**Authors:** Zeynep DAL, Başak ARU

**Affiliations:** 1 6th Phase Student, Faculty of Medicine, Yeditepe University, İstanbul, Turkiye; 2 Department of Immunology, Faculty of Medicine, Yeditepe University, İstanbul, Turkiye

**Keywords:** Colorectal cancer, apoptosis, pyroptosis, NLRP3 inflammasome, DNA content analysis

## Abstract

**Background/aim:**

Colorectal cancer (CRC) is one of the most common cancers worldwide. Many factors such as stress, lifestyle, and dietary habits are known to play a role in the initiation and progression of the disease. Herbal therapeutic agents including curcumin can hold a great potential against cancer treatment; however, their efficacy on CRC is still under investigation. Herein, we evaluated the anticancer mechanism of curcumin on four different CRC cell lines.

**Materials and methods:**

Cells were treated with curcumin for 24, 48 and 72 h, and IC_50_ doses for each cell line were calculated. Mechanistic studies were conducted with the lowest IC_50_ dose determined for each cell line by evaluating apoptosis and necrosis, cell division, and NLRP3-mediated pyroptosis.

**Results:**

Curcumin treatment significantly decreased viability while increasing the SubG1 phase in all cell lines tested, indicating apoptosis is the main programmed cell death pathway activated upon curcumin treatment in CRC. In terms of pyroptosis, components of NLRP3 inflammasome were found to be elevated in SW480 and HCT116 cell lines, although to a lesser extent in the latter, and NLRP3 inflammasome activation was not observed in LoVo and HT29 cells.

**Conclusion:**

Our results reveal that while curcumin effectively induces apoptosis, its effects on NLRP3-inflammasome mediated pyroptosis vary. Our results underline the need for further research focusing on the other inflammasome complexes to confirm the differential effects of curcumin on CRC.

## 1. Introduction

Colorectal cancer (CRC) is the third most common cancer and ranks as the second leading cause of cancer-related deaths worldwide [1]. More than 2.2 million new cases and 1.1 million deaths, mostly caused by metastasis, are expected by 2030 [2, 3]. CRC is defined as the abnormal growth of cells in tissues of the colon and rectum; diagnosis and prognosis of the disease rely on various parameters [4–11]. Flavonoids, a family of polyphenols, are commonly found in fruits and vegetables and are proven to show antiinflammatory properties, moreover, their dietary intake may reduce the risk of CRC development [12–15].

Inflammasomes, multimeric protein complexes, play an important role in the regulation of innate immune responses and consist of sensor proteins, inflammatory caspases, and in some cases, an adapter protein that connects the two, and their activation results in inflammatory cell death, pyroptosis [16, 17]. The most studied inflammasome complex, NLR family pyrin domain containing 3 (NLRP3), is shown to be induced by damage-associated molecular patterns (DAMPs) and pathogen-associated molecular patterns (PAMPs) leading to the activation of caspase-1 by cleavage and subsequent release of proinflammatory cytokines interleukin 1 beta (IL-1β) and interleukin 18 (IL-18) [18]. The roles of inflammasome complexes and their products, IL-1β and IL-18, on the intestinal mucosal homeostasis and inflammation are already noted in the existing literature [19, 20] and they have also shown to be involved in carcinogenesis. However, the role of NLRP3 complex in cancer remains controversial since it can both induce or suppress cancer growth [21, 22].

Our current study aims to investigate the mechanistic effect of curcumin, an anticancer and antiinflammatory flavonoid derived from turmeric, on four different CRC cell lines; HCT116, SW480, LoVo, and HT29, bearing different oncogenic mutations. Curcumin has previously been shown to inhibit CRC growth by blocking cell cycle progression and promoting apoptosis [23], but its impact on the NLRP3 inflammasome complex in CRC cell lines had not been studied. For this purpose, we first investigated the IC_50_ doses of the compound on all cell lines and evaluated respective doses’ impact on cellular DNA content, as well as their ability to initiate programmed cell death pathways apoptosis and NLRP3-mediated pyroptosis.

## 2. Materials and methods

### 2.1. Cell culture and determination of cytotoxicity

HCT116 and LoVo cells were cultured in DMEM/F12 (Pan Biotech, #P04-41500), HT29 cells were cultured in McCoy’s 5A (Cytiva HyClone, #SH30200.01) and SW480 cells were cultured in DMEM (Thermo Fisher Scientific, #41966029) media, all supplemented with 10% foetal bovine serum (Sigma Aldric, #F7524) and 100 U/mL penicillin-100 μg/mL streptomycin antibiotic mixture (Thermo Fisher Scientific, #15140122) at final concentrations. The cell lines abovementioned were chosen to represent different oncogenic mutations that are observed in CRC cases: HCT116 and LoVo cells both have KRAS^G13D^ mutations in addition to the KRAS^A14V^ mutation observed in LoVo cell line [24]; HCT116 cell line harbors a hotspot mutation in the exon 20 of PIK3CA (PIK3CA^H1047R^) [25, 26]; KRAS^G12V^, TP53^R273H^ and TP53^P309S^ mutations are observed in SW480 cells [24, 27]; and HT29 harbors BRAF^V600E^, PIK3CA^P449T,^ and TP53^R273H^ mutations [28]. All cells were passaged at 80% confluency by trypsin-EDTA solution (Thermo Fisher Scientific, #25200056). All centrifugations were performed at 300×G for 5 min unless stated otherwise. For determining cytotoxicity, 5 × 10^3^ cells per well were seeded into 96-well cell culture plates as triplicates and incubated overnight to allow attachment followed by the treatment with curcumin at doses of 100, 50, 25, 5 and 1 μM. Viability was measured at 24, 48, and 72 h by MTS assay (Abcam, #ab197010), which relies on the reduction of the reagent by active mitochondrial reductases in viable cells to yield a colored formazan product [29]. Absorbance at 490 nm was measured by a spectrophotometer (Epoch, BioTek Instruments), and cytotoxicity was calculated by dividing the absorbance of the test group by the control group and then multiplying by 100 to obtain the viability percentage upon treatments at different doses of curcumin abovementioned. Studies regarding the anticancer mechanisms of curcumin were conducted at 72 h, where the lowest IC_50_ values were obtained for all cell lines.

### 2.2. Annexin V/propidium iodide staining

Viability, apoptosis, and necrosis were evaluated by Annexin V/propidium iodide (PI) staining. For this purpose, cells were seeded into 60-mm Petri dishes as 5 × 10^5^ cells per dish and incubated overnight to allow attachment. Cells were then treated with curcumin at respective IC_50_ doses for 72 h. For Annexin V/PI staining, cells were detached by trypsinization, washed once with Dulbecco’s phosphate-buffered saline solution (DPBS, Thermo Fisher Scientific, #14190144), and suspended in 1X ice cold Annexin V Binding Buffer (BioVision Inc., #1006) followed by labelling with 1 μL Annexin V-FITC reagent (BioVision Inc., #1001) and 1 μL PI solution (Thermo Fisher Scientific, #P3566, diluted to 250 μg/mL in DPBS) by incubating for 10 min under dark conditions at room temperature. 2.5 × 10^4^ cells per test were read with Navios flow cytometry system (Beckman Coulter Inc.) and results were analyzed by Kaluza software (version 2.1). All experiments were performed as triplicates.

### 2.3. DNA content analysis

DNA content analysis was performed by Cell Cycle Kit (Beckman Coulter Inc., #C03551). Cells were seeded as 5 × 10^5^ cells/60 mm dish and incubated overnight for attachment. After incubation with curcumin at respective IC_50_ doses for 72 h, cells were detached by trypsinization, washed once with DPBS (Thermo Fisher Scientific, #14190144), and fixed with ice-cold 70% ethanol by adding dropwise followed by incubation at 4 °C for an hour. Tubes were stored at −20 °C overnight, and ethanol was discarded by centrifuging cells at 400×G for 5 min. Pellet was suspended in 1 mL Cell Cycle Kit reagent, and tubes were incubated at room temperature in dark for 30 min. DNA content was analyzed by the DxFLEX flow cytometry system (Beckman Coulter Inc.). Analyses were performed with ModFit LT software (ver. 4.0; Verity Software House).

### 2.4. RNA isolation and qRT-PCR

Expression levels of NLRP3, PYCARD, caspase-1, IL-18, and IL-1β were evaluated with quantitative reverse transcription PCR (qRT-PCR). Total RNAs of cells treated with curcumin at respective IC_50_ doses for 72 h were isolated by TRIzol Plus RNA Purification Kit (Thermo Fisher Scientific, #12183555), which couples the phenol-chloroform lysis with silica column-based isolation. RNA concentration and purity were determined by a spectrophotometer (Epoch, BioTek Instruments). Five hundred nanograms of total RNA was transcribed using the High-Capacity cDNA Reverse Transcription Kit (Thermo Fisher Scientific, #4368814) according to the manufacturer’s instructions. qRT-PCR was performed using KiCqStart SYBR Green qPCR ReadyMix (Sigma Aldrich, #KCQS02) on StepOnePlus Real-Time PCR System (Applied Biosystems) as triplicates. Untreated cells were used as control. Target mRNA quantities were normalized to glyceraldehyde 3-phosphate dehydrogenase (GAPDH), a suitable control gene thanks to its low variance profile unless mitogenic stimulation is present [30]. Relative gene expressions were calculated by the 2^−ΔΔCt^ method. The oligonucleotides used for qRT-PCR are provided in Table 1.

### 2.5. Statistical analysis

Statistical analyses were conducted with GraphPad Prism software (Version: 8.0). The effects of curcumin on viability, early apoptosis, late apoptosis, and necrosis were evaluated with one-way analysis of variance (ANOVA) test followed by Tukey’s multiple comparison tests while unpaired t-test was used for analyses regarding DNA content and qRT-PCR. All tests were conducted in triplicates. p values lower than 0.05 were considered statistically significant.

## 3. Results

### 3.1. Determination of cytotoxicity

In vitro cytotoxicity of curcumin on CRC cell lines HCT116, SW480, LoVo, and HT29 was evaluated with MTS assay at 24, 48, and 72 h where IC_50_ values gradually decreased in a time-dependent manner and are presented in Table 2. Since the lowest IC_50_ values were obtained on the 72nd hour for all cell lines, further studies were conducted with the respective IC_50_ value of curcumin regarding each cell line at this timepoint.

### 3.2. Annexin V/propidium iodide staining

**Figure 1:**
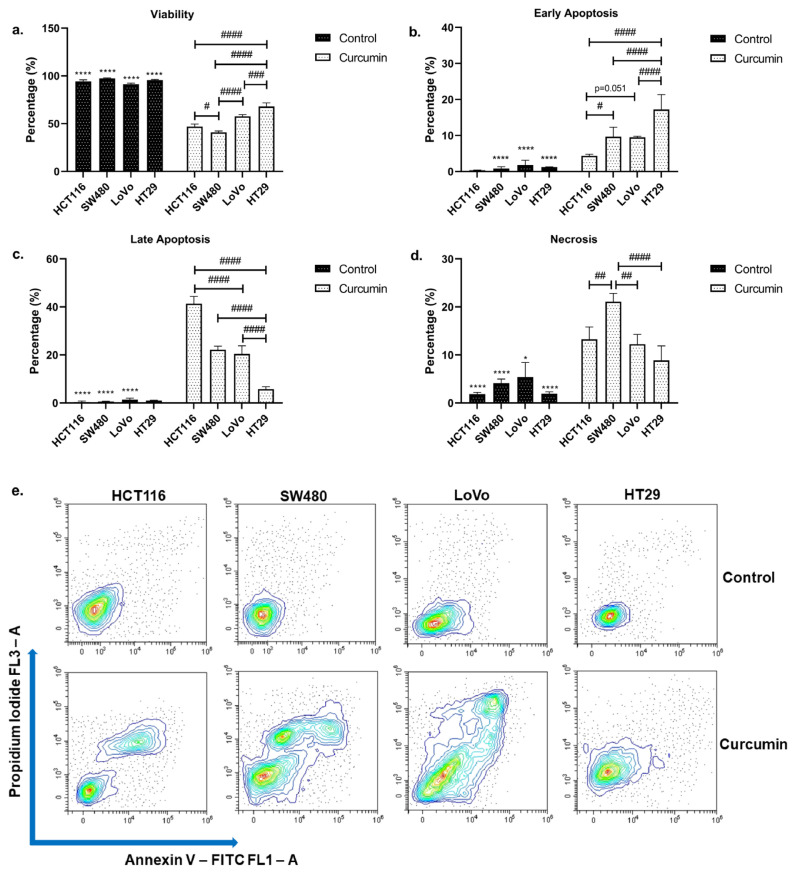
Curcumin decreases viability significantly in the CRC cell lines while inducing apoptosis and necrosis. Bar graphics indicating **(a)** viability, **(b)** early apoptosis, **(c)** late apoptosis and **(d)** necrosis. * indicates differences between respective control and test groups; # indicates differences between treatment groups. **(e)** Representative flow cytometry plots. *p < 0.05, **p < 0.01, ***p < 0.001, ****p < 0.0001; #p < 0.05, ##p < 0.01, ###p < 0.001, ####p < 0.0001.

Annexin V/PI staining revealed that curcumin treatment significantly decreased viability in all cell lines (p < 0.05), underlining the anticancer effect of the compound on CRC (Figures 1a–1e). Along with the significantly decreased viability rates (Figure 1a), early apoptosis (Figure 1b), late apoptosis (Figure 1c), and necrosis (Figure 1d) rates were significantly increased in SW480 and LoVo cells (p < 0.05), indicating curcumin is an effective inducer of apoptosis and necrosis in these cell lines. Similarly, curcumin led to a significant decrease in viability (p < 0.05) (Figure 1a) without promoting early apoptosis (p > 0.05) in HCT116 cells (Figure 1b). The decrease in viability was accompanied by significant increases in late apoptosis (Figure 1c) and necrosis (Figure 1d) in this cell line (p < 0.05). HCT116 also had the highest late apoptosis rates among all cell lines tested (p < 0.05). Thus, it can be concluded that curcumin promotes apoptosis and necrosis simultaneously in HCT116, SW480, and LoVo cell lines when administered at respective IC_50_ values. Curcumin significantly decreased viability in HT29 cells (p < 0.05) (Figure 1a) while promoting early apoptosis (p < 0.05) (Figure 1b), though the increase in late apoptosis was found to be nonsignificant (p > 0.05) (Figure 1c). On the other hand, necrosis rates were significantly increased (p < 0.05) in this cell line (Figure 1d). A comparison between cell lines also revealed that HT29 cells had the highest early apoptosis (p < 0.05) and lowest late apoptosis rates among all cell lines tested (p < 0.05). Representative flow cytometry histograms are presented in Figure 1e.

### 3.3. DNA content analysis

**Figure 2:**
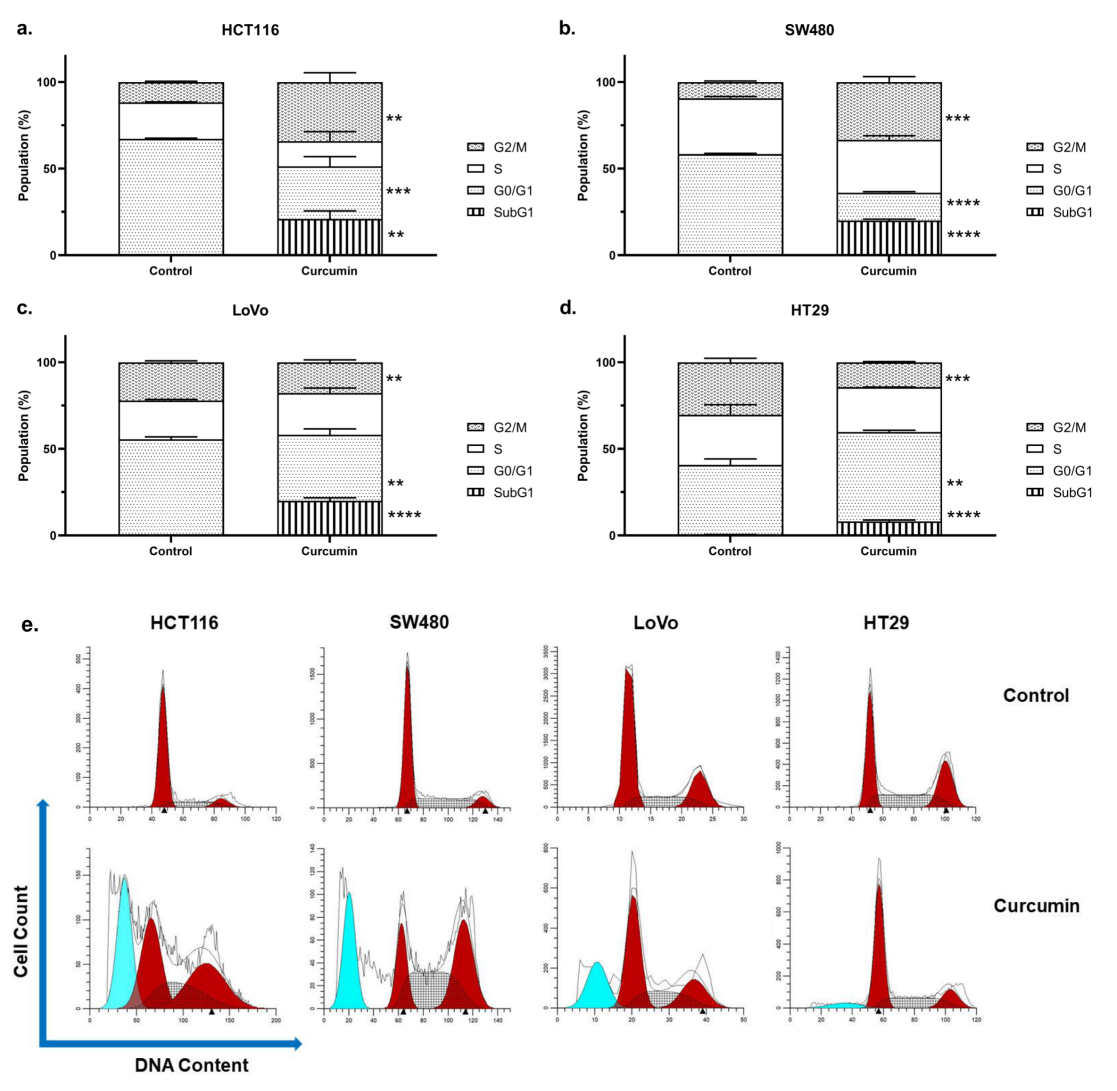
Curcumin increases SubG1 phase in all cell lines and promotes cell cycle arrest in a cell line-dependent manner. Bar graphics indicate cell cycle distribution for **(a)** HCT116, **(b)** SW480, **(c)** LoVo, and **(d)** HT29. * indicates differences between respective control and test groups. **(e)** Representative flow cytometry histogram plots. **p < 0.01, ***p < 0.001, ****p < 0.0001.

Curcumin significantly increased the SubG1 phase in all cell lines (p < 0.05), underlining the apoptotic effect of the compound. Comparisons between cell lines revealed that HT29 cells upon curcumin treatment had significantly lower SubG1 populations compared to LoVo, HCT116, and SW480 cells (p < 0.05) (Figures 2a–2e). In addition to SubG1 phase, the G2/M phase was significantly increased in HCT116 (Figure 2a) and SW480 (Figure 2b) cell lines (p < 0.05), which was accompanied by a decrease in G0/G1 phase (p < 0.05). Although curcumin induced G2/M phase arrest in both, no difference between SW480 and HCT116 cells in terms of G2/M phases was observed (p > 0.05). Similarly, G0/G1 phase was significantly decreased in LoVo cells (p < 0.05); however, cell cycle arrest was not observed in this cell line (Figure 2c). On the other hand, G1/G0 phase was increased in HT29 cells along with a significant decrease in the G2/M phase (p < 0.05) (Figure 2d). Altogether, these data indicate that when administered at respective IC_50_ doses of each cell line, curcumin leads to arrest during different phases of the cell cycle in different CRC cell lines. Representative flow cytometry histograms are presented in Figure 2e.

### 3.4. Gene expression analysis

**Figure 3:**
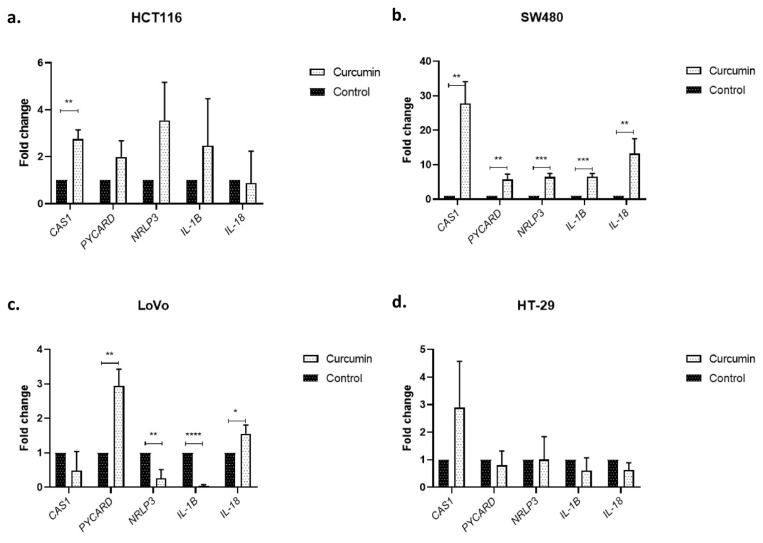
Curcumin induces NLRP3 inflammasome complex in a cell line-dependent manner. Bar graphics indicate relative gene expression levels in terms of NLRP3 inflammasome components in **(a)** HCT116, **(b)** SW480, **(c)** LoVo, and **(d)** HT29 cell lines. * indicates differences between respective control and test groups. *p < 0.05, **p < 0.01, ***p < 0.001, ****p < 0.0001.

Curcumin led to a significant increase in caspase-1 (p < 0.01) in HCT116 cells, yet the increases in NLRP3, PYCARD, and IL-1β were not significant (p > 0.05) (Figure 3a). On the other hand, in SW480 cell line, all parameters of the NLRP3 inflammasome complex evaluated (caspase-1 and IL-18 p < 0.01; PYCARD, NLRP3, and IL-1β p < 0.001) were significantly enhanced (Figure 3b). These findings suggest that curcumin can promote NLRP3-mediated pyroptosis in SW480 and HCT116 cell lines, although to a lesser extent in the latter. On the contrary, curcumin repressed NLRP3 (p < 0.01) and IL-1β (p < 0.001) while increasing PYCARD and IL-18 levels (p < 0.01) without altering caspase-1 expression (p > 0.05) in LoVo cells, indicating at least NLRP3-mediated pyroptosis is not involved in the anticancer efficacy of curcumin on this cell line but increased PYCARD and IL-18 levels may suggest that another inflammasome may be involved in this process (Figure 3c). NLRP3 inflammasome activation was not observed in the HT29 cell line as none of the parameters were shown to be increased upon curcumin treatment in this cell line (p > 0.05), which may be attributed to the HT29 cell line’s distinct mutation profile (Figure 3d).

## 4. Discussion

The search for herbal therapeutic agents to combat cancer dates back to the 1950s, the discovery of vinca alkaloids while currently, many molecules in clinical practice such as Taxol, Vinblastine or Vincristine, Irinotecan, Camptothecin, and their analogues are plant-derived [31, 32]. The main polyphenol extracted from *Curcuma longa*, curcumin, has a wide range of therapeutic potential in the treatment of various diseases including cancer: 37% of all studies involving curcumin focus on the anticancer effect of the compound, and this activity is mainly attributed to its antioxidant and antiinflammatory properties since disruptions of inflammatory pathways play role in carcinogenesis [33]. Curcumin can regulate several signaling pathways involved in cancer development and is regarded as an effective anticancer compound, either alone or in combination with other drugs [33].

When considering curcumin in CRC treatment, in vitro studies indicated that the compound could lead to cell cycle arrest at G0/G1 and G2/M phases and promote apoptosis by regulating various molecular pathways, while its chemopreventive effects were mainly reported in vivo [23]. In terms of in vitro studies, curcumin has been suggested to inhibit cell growth and induce apoptosis in a mitochondria-dependent fashion in the LoVo cell line while in HCT116 and HT29 cell lines, it inhibited hexokinase II, an enzyme often expressed in aggressive tumors with a high aerobic glycolysis rate [34, 35]. When considering SW480 cells, Yavuz-Türel et al. reported increased apoptosis rates upon curcumin treatment, while Dou et al. suggested that instead of promoting apoptosis, the compound inhibits cellular proliferation via WNT/catenin pathway [36, 37]. Compatible with the literature, in our study, curcumin promoted apoptosis in SW480, LoVo, and HCT116 cell lines, while the latter had the highest late apoptosis rates among all cell lines tested, indicating apoptosis is the major regulated cell death pathway in HCT116 cells upon curcumin treatment. On the other hand, early apoptosis was found to be considerably higher in the HT29 cell line, suggesting higher doses of curcumin or longer incubation periods with the compound may be required for apoptotic response in this cell line. In addition to apoptosis, necrosis was found to be increased in all cell lines tested: as this pathway can be employed under apoptosis-deficient conditions [38], this finding indicates that necroptosis should also be investigated to determine if the compound can be a treatment option for cancers resistant to apoptosis.

In our study, apoptosis in CRC cells was also confirmed with DNA content analysis where increased SubG1 phases in all cell lines were observed. Similar to Annexin V/PI staining, the highest SubG1 ratio was observed in HCT116 cells, underlining the apoptotic effect of curcumin on this cell line. Increased SubG1 phase was accompanied by G2/M-phase arrest in HCT116 and SW480 cells and G1/G0-phase arrest in HT29 cell lines; even though S-phase was increased in LoVo cells, this alteration was not significant and cell cycle arrest was not observed in this cell line. Similarly, Tang and Yang reported G2/M-phase arrest in HCT116 and SW480 cells upon curcumin treatment while Rahim et al. indicated G0/G1-phase arrest in HT29 cells when treated with curcumin analogues [39, 40]. In brief, extensive studies regarding the mechanistic effects of curcumin in CRC have underlined that apoptosis and cell cycle arrest are the main events mediating the compound’s anticancer properties while its effects on pyroptosis, inflammatory cell death, which involves inflammasome activation and maturation of proinflammatory cytokines, IL-1β and IL-18, remained elusive [41].

Pyroptosis can either be induced via activation of multimeric inflammasome complexes assembled in response to DAMPs or PAMPs or directly by lipopolysaccharide-bound caspase-4, -5, and -11 [42, 43]. Inflammasome assembly by homooligomerization of intracellular sensor proteins is followed by recruitment of the adaptor protein containing CARD through Pyrin domains. This complex interacts with procaspase-1 to activate it through self-cleavage which results in IL-1β and IL-18 maturation. Among all inflammasome complexes, NLRP3 has been directly associated with inflammatory diseases and indicated to be involved in carcinogenesis [43]. NLRP3 inflammasome-related genes have been proven to be dysregulated in various cancers and these alterations may either lead to favorable or detrimental results. There are also reports in the literature that reveal inflammasome activation in tumor-infiltrating immune cells but not the tumor stroma [22, 44]. Investigating the link between epithelial-to-mesenchymal transition (EMT) markers and NLRP3 inflammasome in CRC in vitro, Wang et al. revealed enhanced NLRP3 expression during EMT in HT29 and HCT116 cells, while active caspase-1 and PYCARD were not detected, indicating inflammasome-independent NLRP3 is involved in EMT in CRC [45]. Cambui et al. suggested that genetic variants leading to increased NLRP1 or NLRP3 activation along with decreased IL-18 levels have detrimental effects on CRC prognosis [46]. The protective role of IL-18 in CRC is an interesting finding since IL-18 expression has been studied in various cancers and is generally linked with poor prognosis [47]. However, the reduction or absence of this cytokine in CRC tissues suggest a protective role [48]. On the other hand, increased IL-1β levels, another product of activated NLRP3 inflammasome complex, were detected in CRC tissues and associated with cetuximab resistance [20 ,49]. Thus, it can be concluded that IL-1β and IL-18, two products of NLRP3 inflammasome likely exert contradictory effects in CRC. In our study, curcumin successfully activated NLRP3-dependent pyroptosis in the SW480 cell line while longer incubation periods or higher doses may be required for HCT116 cells; the significant increase in PYCARD and IL-18 levels in the latter may also suggest the involvement of an another inflammasome complex rather than NLRP3, or an inflammasome-independent action of PYCARD [50]. In the HT29 cell line, none of the NLRP3 inflammasome components were observed to be increased, indicating curcumin does not promote NLRP3-dependent pyroptosis in this cell line.

It is worth mentioning that the cell lines used in this study differ in terms of oncogenic mutations their genomes harbor. Previously, B-Raf proto-oncogene, serine/threonine kinase (BRAF), and phosphatidylinositol-4,5-bisphosphate 3-kinase catalytic subunit alpha (PIK3CA) mutations were linked with apoptosis resistance [51, 52]. BRAF mutations are observed in approximately 10% of all patients diagnosed with CRC and lead to unique morphological, clinical, and therapeutic characteristics [53]; in addition, they were reported to be linked with the right-sided tumor localization and antiepithelial growth factor receptor (EGFR) therapy resistance [54]. Mutations of PIK3CA have been reported in 10%–20% of CRC cases and shown to be largely associated with KRAS mutations [55]. Approximately 30%–40% of CRC cases bear KRAS mutations, which are associated with lower apoptotic index, poorer survival, enhanced aggressiveness, and treatment resistance [56–58]. According to a study of Dinu et al., in stage I and II CRC patients, mutations in codon 13 of KRAS leads a statistically significant shorter survival rate than for those with wild type [57]. Similar to BRAF mutations, KRAS mutations are also associated with right-sided CRC, which have a significantly worse 5-year overall survival rate in comparison with left-sided CRC [59]. KRAS-mutant tumors are also reported to respond poorly to anti-EGFR therapy [54]. Investigating the role of differential KRAS mutations in the response to anti-EGFR therapy in vitro, Kumar et al. revealed that cetuximab and panitumumab exert moderate success on HCT116 and LoVo cell lines in a dose-dependent manner, while SW480 cells were shown to be resistant to both [60]. However, when considering double TP53 mutation, which the SW480 cell line has, the success of anti-EGFR therapies may not be solely linked to the KRAS mutation but also to the presence of functional p53 protein as SW480 cells were proven to express mutated p53 protein 20-fold higher compared to their TP53^wt^ counterpart LoVo cells, though the R273H/P309S-mutated p53 protein in SW480 is capable of activating another cell cycle control protein p21(Cip1/WAF1) [27]. On the other hand, the relatively distinct mutation profile of the HT29 cell line might be one of the underlying causes of the ineffectiveness of curcumin on the NLRP3 inflammasome complex in this cell line.

When taken together, curcumin had differential effects on four different CRC cell lines when administered at respective IC_50_ doses such as promoting apoptosis, necrosis, and NLRP3-dependent pyroptosis in SW480 and to a lesser extent in HCT116 cells, while apoptosis and necrosis were the predominant cell death pathways activated in LoVo cells. The significant increase in early apoptosis in the HT29 cell line may suggest that higher doses of curcumin or longer incubation periods may be required for a more prominent effect, and the respective IC_50_ dose of this flavonoid leads to cell cycle arrest in a 72-h period for this cell line. However, it should be noted that pyroptosis can be modulated with a wide array of different inflammasome complexes [16], and thus, more research involving the activation of other inflammasomes is required for unveiling the pyroptotic effect of curcumin on CRC. Besides its anticancer properties, curcumin can also exert chemopreventive effect by downregulating the NLRP3 inflammasome complex in a context-dependent manner. In a dextran sulfate sodium (DSS)-induced colitis model, curcumin was shown to alleviate inflammation in the colon and attenuate symptoms of DSS-induced colitis [61]. Moreover, curcumin pretreatment before lipopolysaccharide (LPS) challenge diminished DSS-induced IL-1β release by inhibiting NLRP3 inflammasome [61]. Previously, curcumin has been shown to inhibit NLRP3 inflammasome activation in macrophages both in vitro and in vivo [62, 63]. These data indicate that when considering patients with chronic colitis are more susceptible to developing CRC [4], curcumin can also be beneficial in terms of preventing tumorigenesis by inhibiting the NLRP3 inflammasome complex in macrophages at the inflammation site, and can hamper the inflammation.

The inflammasome activation relies on the interaction between the components of the inflammasome complex. Other strategies targeting the assembly of the inflammasome complexes rather than investigating components’ protein or gene expression levels individually may provide a better insight into the pyroptosis-mediated anticancer efficacy of curcumin. Moreover, activation of programmed cell death pathways is a phenomenon involving multiple pathways simultaneously, and other cell death pathways should be considered to evaluate the anticancer efficacy of curcumin [64]. Finally, the bioavailability of curcumin is known to be quite limited due to its low dissolvability, intestinal absorbability, and fast metabolism; novel drug delivery strategies such as the development of nanocapsules or sublingual pills will contribute to its administration as a therapeutic supplement [65].

## Figures and Tables

**Table 1:** Primers used in this study.

	Forward	Reverse
GAPDH	CGACCACTTTGTCAAGCTCA	AGGGGTCTACATGGCAACTG
IL1β	GCATCCAGCTACGAATCTCC	CGTGCACATAAGCCTCGTTA
IL18	TTGTCTCCCAGTGCATTTTG	TGCCACAAAGTTGATGCAAT
PYCARD	TGACGGATGAGCAGTACCAG	AGGATGATTTGGTGGGATTG
NLRP3	CGAGGGGTCAGACAGAGAAG	TTCCTGGCATATCACAGTGG
Caspase-1	GGGTGCTGAACAAGGAAGAG	TAGCTGGGTTGTCCTGCACT

**Table 2:** IC_50_ values of curcumin for colon cancer cell lines HCT116, SW480, LoVo and HT29 on 24, 48, and 72 h.

Cell Lines	IC_50_ (μM)		
	24 h	48 h	72 h
**HCT116**	63.84	20.32	19.05
**SW480**	38.77	19.26	15.63
**LoVo**	49.84	18.42	13.53
**HT29**	54.46	28.16	22.10
